# Necroptosis-Related LncRNA Signatures for Prognostic Prediction in Uterine Corpora Endometrial Cancer

**DOI:** 10.1007/s43032-022-01023-9

**Published:** 2022-07-19

**Authors:** Zhiheng Lin, Weisen Fan, Xiaohui Sui, Juntao Wang, Junde Zhao

**Affiliations:** 1 Shandong University of Traditional Chinese Medicine, Jinan, 250014 Shandong China; 2 Department of Orthopedic Surgery, Qilu Hospital of Shandong University, Jinan, 250012 Shandong China

**Keywords:** Necroptosis, UCEC, LncRNA, Prognostic, Signature

## Abstract

Necroptosis is one of the common modes of apoptosis, and it has an intrinsic association with cancer prognosis. However, the role of the necroptosis-related long non-coding RNA LncRNA (NRLncRNAs) in uterine corpora endometrial cancer (UCEC) has not yet been fully elucidated at present. Therefore, the present study is designed to investigate the potential prognostic value of necroptosis-related LncRNAs in UCEC. In the present study, the expression profiles and clinical data of UCEC patients were downloaded from TCGA database to identify the differentially expressed NRLncRNAs associated with overall survival. A LncRNA risk model was constructed via Cox regression analysis, and its prognostic value was evaluated. We have also further evaluated the relationships between the LncRNA features and the related cellular function, related pathways, immune status, and immune checkpoints m6A-related genes. Seven signatures, including PCAT19, CDKN2B-AS1, LINC01936, LINC02178, BMPR1B-DT, LINC00237, and TRPM2-AS, were established to assess the overall survival (OS) of the UCEC in the present study. Survival analysis and ROC curves indicated that the correlated signature has good predictable performance. The normogram could accurately predict the overall survival of the patients with an excellent clinical practical value. Enrichment analysis of gene sets indicated that risk signals were enriched in several immune-related pathways. In addition, the risk characteristics were significantly correlated with immune cells, immune function, immune cell infiltration, immune checkpoints, and some m6A-related genes. This study has identified seven necroptosis-related LncRNA signatures for the first time, providing a valuable basis for a more accurate prognostic prediction of UCEC.

**Supplementary Information:**

The online version contains supplementary material available at 10.1007/s43032-022-01023-9.

## Introduction

Uterine corpora endometrial cancer (UCEC) is one [1] of the common gynecological malignancies. In recent years, the incidence and mortality of UCEC are increasing year by year [2, 3]. Currently, transvaginal ultrasound with endometrial tissue biopsy is the main method of diagnosing endometrial cancer. In cases of uncertainty, hysteroscopy is feasible [4]. At present, the surgery, adjuvant radiotherapy, chemotherapy, and other treatment methods [2, 5] are the main methods to treat the UCEC. However, the UCEC is easy to metastasize [5] via lymph nodes, and the disease is prone to relapse [6] after treatment. Therefore, it is essential to find out new prognosis-related biosignatures to guide the clinical treatment of UCEC patients.

Necroptosis is a programmed cell death mediated [7–10] by the receptor-interacting protein kinase-3 (RIPK3) and its substrate mixed lineage kinase-like (MLKL). Resistance to apoptosis has been reported to be one of the main marks of tumors [11]. Thus, the emergence of mature immune patterns directed against necroptosis has great implications for cancer treatment. Low expression of RIPK3 improves tumor necrosis factor–mediated cell damage. The kinase activity of RIPK3 is critical [12, 13] for the process of cell necrosis. Long non-coding RNA (LncRNAs) refers to the RNA [14, 15] that is longer than 200 nucleotides with no protein-coding capability. Genome-wide association studies of tumor samples have identified a large number of LncRNAs [16] associated with various types of cancers. For example, many LncRNAs have been shown to be potential biosignatures and targets [17] for the diagnosis and treatment of cancer. In addition, immune-related [12, 13] LncRNAs may be disorderly expressed in tumors and significantly correlated with the immune cell infiltration [18].

**Fig. 1:**
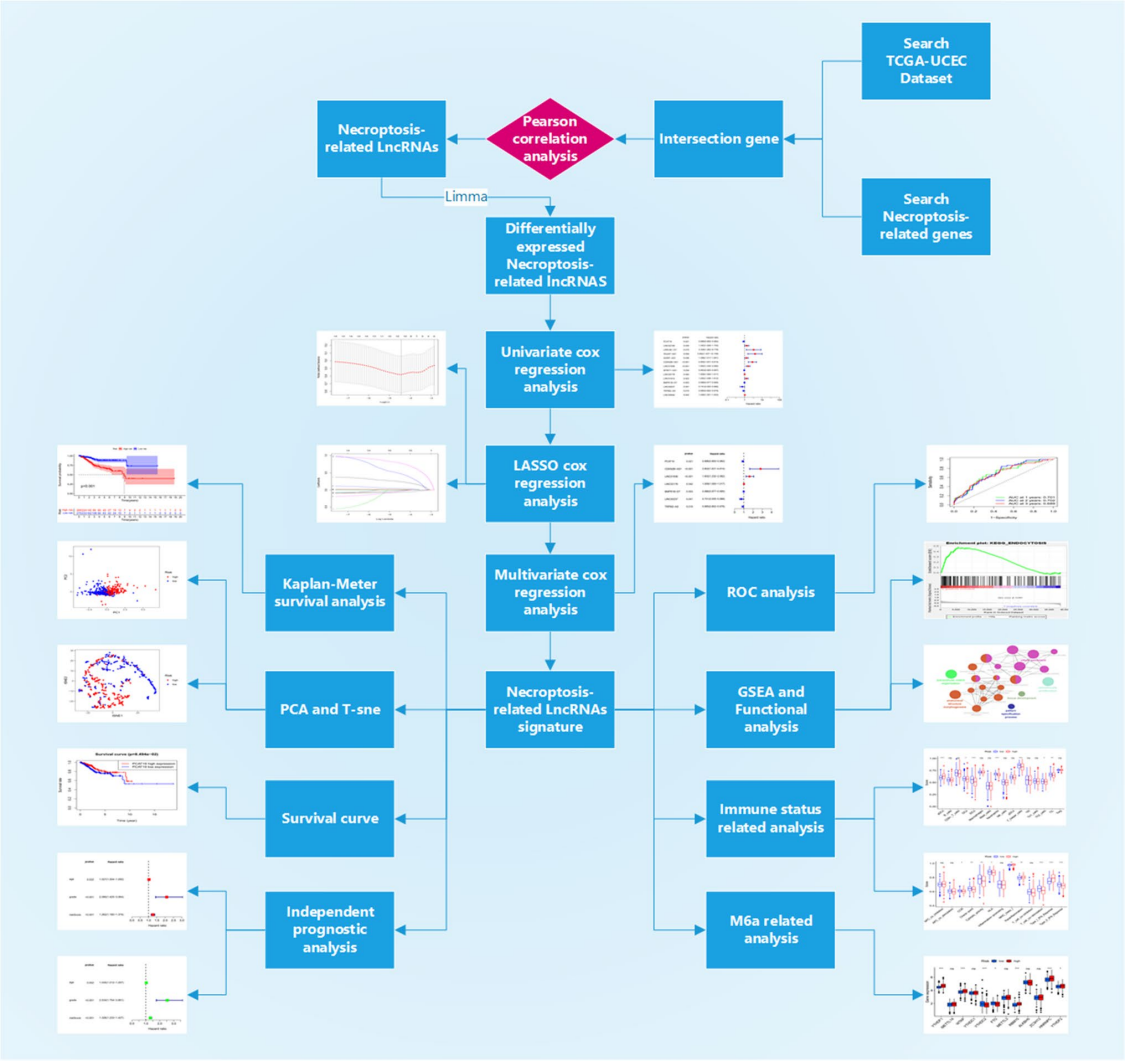
Flow chart of the entire study. Throughout the workflow, risk score prognostic models for necroptosis-related LncRNAs (NRLncRNAs) were constructed in UCEC samples by univariate cox regression analysis, lasso regression, and multivariate cox regression analysis. A series of correlation and prognostic analyses were performed on patients in the high- and low-risk score groups, and a final screen of seven prognostically relevant NRLncRNAs was performed

Therefore, it is significantly important for the prognosis in UCEC by finding out the key LncRNAs closely related to the necroptosis. In this paper, we applied the bioinformatic methods to identify specific biosignatures related to the prognosis of UCEC, providing new perspectives for the prognosis of UCEC. See Fig. 1 for a flow chart of our study. Throughout the workflow, we constructed risk score prognostic models for necroptosis-related LncRNAs (NRLncRNAs) in UCEC samples by univariate cox regression analysis, lasso regression, and multivariate cox regression analysis. A series of correlation and prognostic analyses were performed on patients in the high- and low-risk score groups, and a final screen of seven prognostically relevant NRLncRNAs was performed.

## Materials and Methods

### Data Sources

We obtained RNA sequence data (FPKM values) and their corresponding clinical features from the tumor tissues of 552 UCEC patients and normal tissues of 23 people in The Cancer Genome Atlas (TCGA) database (https://portal.gdc.cancer.gov/repository), as well as extracted and standardized these data using R software (4.1.1) [19]. One hundred and four genes associated with necroptosis were extracted from previous reviews and shown in the Appendix Table Supplementary S1 [7–9, 11, 20–24]. We intersected the gene information in the expression matrix with the genes related to necroptosis to obtain a new necroptosis-related gene (NRGs) expression matrix. Since the TCGA database is a public access resource, the present study is exempted from the approval of the Ethics Committee.

### Identification of NRG-Related LncRNAs

In order to identify the NRG-related LncRNAs, we used the Pearson correlation to assess the association between the LncRNAs and NRGs. Typically, we used a threshold of *p* < 0.001 and a correlation coefficient > 0.4 to select the NRG-related LncRNAs (NRLncRNAs).

The “limma” package [25] of R software was used to identify differentially expressed necroptosis-related LncRNAs between the tumor tissue and normal tissue. By referring to screening criteria of logFC > 1 and *P* < 0.05, differentially expressed NRLncRNAs were acquired in the tumor tissue and normal tissue.

### Construction and Validation of the Prognostic Risk Model of NRLncRNAs

We combined differentially expressed NRLncRNA expression with survival time using the perl language (perl 5.28.1) to remove samples with incomplete clinical information and zero or negative survival time. Univariate Cox regression analysis was performed using the “survival” package to screen out NRLncRNAs with a prognostic value. In order to prevent overfitting, the least absolute shrinkage and selection operator (LASSO) Cox regression model were then used to narrow the candidate gene range and build the prognostic model. After that, the screened genes were subjected to multivariate Cox regression analysis to obtain the prognostic genes, draw the prognostic NRLncRNA gene-related network, build a risk score model for predicting survival time, and output the risk score coefficient (coef) that was included in the model genes. The established prognostic risk score model was calculated as risk value (risk score) = prognostic NRLncRNA gene expression 1 × coef1 + prognostic NRLncRNA gene expression 2 × coef 2 + … + prognostic NRLncRNA gene expression *n* × coef *n*. Patient risk values were calculated according to the risk scoring model formula. These patients were divided into low-risk and high-risk groups based on the median.

We compared the overall survival (OS) between the high-risk and low-risk groups by Kaplan–Meier analysis and drew HR forest plots and heatmaps for the NRLncRNA prognosis model and survival curves of each prognostic gene. ROC is short for receiver operating characteristic curve. The relationship between various variables and risk values was evaluated using univariate and multivariate independent prognostic analyses to determine whether the risk model could serve as an independent prognostic indicator. The evaluation of the model predictive effect was carried out using the “timeROC” package function of the R software and receiver operating characteristic (ROC) curve. In addition, the principal component analysis (PCA) was performed using the “Rtsne” package function of the R software. Moreover, a t-SNE test was conducted to visualize grouping, thereby exploring the distribution of different groups. Samples were distinguished according to risk score. Risk score distribution maps and survival status plots were drawn using the “pheatmap” package of the R software. Besides, a normograph was established to predict the patient outcomes by applying the “regplot” package of the R software, and the “ggDCA” package of the software was used to make a decision curve to analyze the sensitivity of risk features and other clinical pathological characteristics.

### Construction of mRNA-LncRNA Co-expression Network

In order to prove the correlation between the necroptosis-related LncRNAs and its corresponding mRNAs, a mRNA LncRNA co-expression network was constructed and visualized with Cytoscape software (3.7.2) so as to further demonstrate the degree of correlation between the necroptosis-related LncRNAs and its corresponding mRNAs.

### GSEA

Gene set enrichment analysis (GSEA) is used to detect the relevant pathways and biological processes of high-risk population. Expressed gene sets in low-risk or high-risk populations and signature gene sets collected in the Kyoto Encyclopedia of Genes and Genomes (kegg) database V7.4 were analyzed using the GSEA (4.1.0) software [26]. FDR < 0.05 was defined as statistical significance.

### Functional Analysis of Differential Genes in High- and Low-Risk Groups

Patients were divided into high-risk and low-risk groups according to the necroptosis-related prognostic model for differential expression analysis of genes using the limma package in order to explore the possible causes of survival differences between the high- and low-expression groups. Setup filtering conditions: logFC > 1. The FDR (BH)-corrected threshold was P.adj < 0.05. Differentially expressed genes were imported into the ClueGO plugin in Cytoscape software (3.7.2) for functional analysis and setup threshold *P* < 0.05.

### Immune Correlation Analysis

We evaluated the activity of immune cells based on FIRLS features in the high-risk and low-risk populations using CiberSort [27, 28], TIMER [29], MCP-COUNTER (MCP-COUNTER) [30], QUANTISEQ [31], and XCell [32], as well as estimated proportion of immune cells to cancer cells (EPIC) [33] and so on algorithms. Meanwhile, single-sample gene set enrichment analysis (ssGSEA) was conducted using the “gsva” package of the R software in order to assess the infiltration scores of 16 immune cells and the activity of 13 immune pathways. Furthermore, the expression levels of immune checkpoint-related genes may be associated to the therapeutic response of immune checkpoint inhibitors. The relationship between risk scores and immune checkpoints was discussed by examining differences in gene expression levels between the high-risk and low-risk populations.

### Correlation Analysis of m6a Gene

Studies have shown that m6A modifications can influence the complexity [34] of cancer progression by modulating the biological function associated with cancer. The m6A-related genes are key regulatory genes of tumor progression. Therefore, we discussed the relationship between the risk scores and m6a-related genes by examining differences in gene expression levels in high-risk and low-risk populations.

## Results

### Identification of Necroptosis-Related Differentially Expressed LncRNAs

Figure 1 illustrates the workflow of this study. Firstly, 4668 LncRNAs were identified in the RNA-SEQ data from UCEC patients based on the latest LncRNA annotation file (see Supplementary S2). These LncRNAs were then analyzed for Pearson correlation with 74 NRGs to yield 459 necroptosis-related LncRNAs (NRLncRNAs). Finally, there were 85 differentially expressed NRLncRNAs identified.

### Construction of Prognostic Gene Risk Models of NRGs

**Fig. 2:**
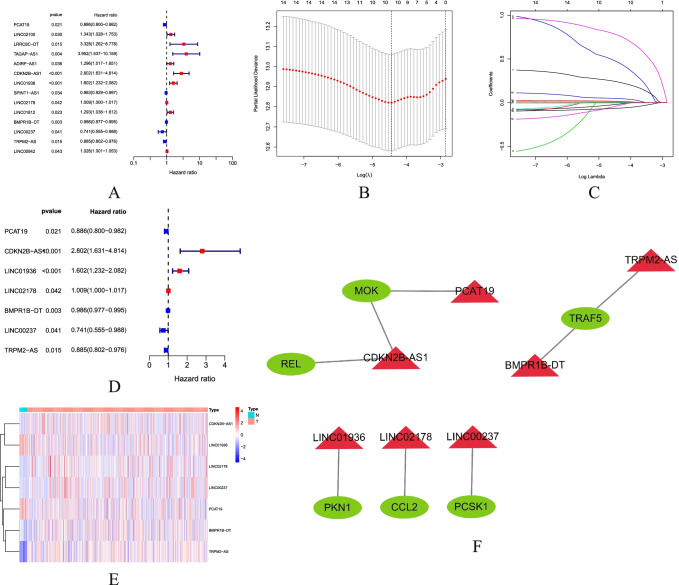
Identification of prognostic necroptosis-related LncRNAs. **A** Univariate cox regression forest plot shows that 14 prognostic-related NRLncRNAs are initially screened. Red indicates that the Hazard ratio (HR) index is greater than 0, blue indicates that the HR index is less than 0; **B**, **C** Lasso-Cox regression analysis shows that 10 RNLncRNAs are good candidates for constructing prognostic characteristics; **D** multivariate cox regression forest plot shows that PCAT19, CDKN2B-AS1, LINC01936, LINC02178, BMPR1B-DT, LINC00237, and TRPM2-AS were prognosis-associated NRLncRNAs and were involved in forming the prognostic model. Red indicates that the HR index is greater than 0, blue indicates that the HR index is less than 0; **E** heatmap of prognostic-related NRlncRNAs. Blue represents low expression of LncRNA, red represents high expression of LncRNA; **F** mRNA-LncRNA co-expression network of prognosis-associated NRlncRNAs and necroptosis-related genes. Green molecules indicate mRNA, red molecules indicate LncRNA

A total of 539 UCEC samples were matched to the corresponding patients with complete survival data. Prognosis-related genes were preliminarily screened using univariate Cox regression analysis. A total of 14 NRLncRNAs were found associated with prognosis (Fig. 2A). Lasso regression analysis and multivariate Cox regression analysis were performed afterwards, and the results showed that seven genes were included in the model (Fig. 2B–D). These seven prognostic NRLncRNAs were PCAT19, CDKN2B-AS1, LINC01936, LINC02178, BMPR1B-DT, LINC00237, and TRPM2-AS, respectively. Based on the best *λ* value, a prognostic risk score model of NRLncRNAs was established: risk score = (− 0.0784 × PCAT19 expression score + (1.0279 × CDKN2B-AS1 expression score) + (0.3622 × LINC01936 expression score) + (0.0099 LINC02178 expression score) + (− 0.0065 × BMPR1B-DT expression score) + (− 0.1754 × LINC00237 expression score) + (− 0. 0801 × TRPM2-AS expression score). Figure 2E shows the heatmap of NRLncRNA expression in the normal group and the tumor group. Figure 2F illustrates the mRNA-LncRNA co-expression network of prognosis-associated NRLncRNAs and necroptosis-related genes.

### Evaluation of Prognostic Risk Score Model

**Fig. 3:**
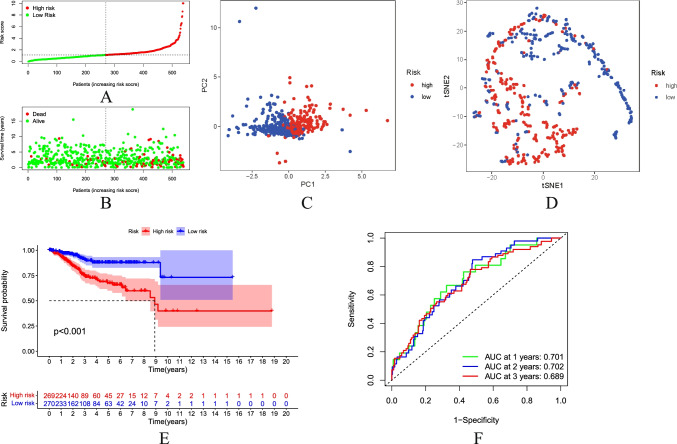
Prognostic analysis of the characteristic model of necroptosis-related LncRNAs. **A** Distribution map of patients in high-risk group and low-risk groups. **B** Patient’s survival status chart. **C** Principal component analysis (PCA) plot. **D** *t*-SNE (*t*-distributed stochastic neighbor embedding) plot. **E** Kaplan–Meier survival curve analysis showed significant differences in OS between low- and high-risk score groups. **F** The AUC of the ROC curve validated the prognostic accuracy of the risk score

**Fig. 4:**
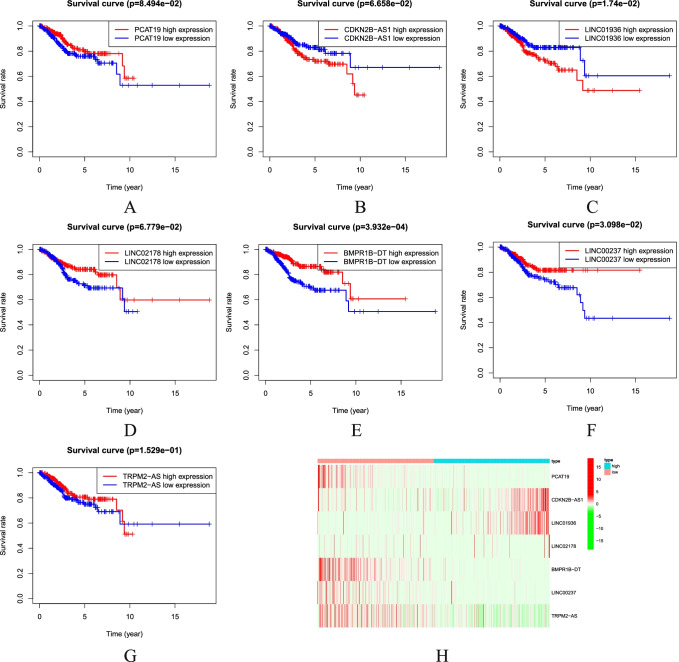
Survival curve of prognosis-associated NRLncRNAs. **A** PCAT19. **B** CDKN2B-AS1. **C** LINC01936. **D** LINC02178. **E** BMPR1B-DT. **F** LINC00237. **G** TRPM2-AS. **H** The heatmap showing the expression of prognosis-related NRLncRNAs between high- and low-risk groups

Through univariate Cox regression analysis, lasso regression analysis, and multivariate Cox regression analysis algorithms, according to the stepwise regression algorithm, the prognosis-related LncRNAs are finally screened from numerous necroptosis-related LncRNAs in order to predict LncRNA molecules that are more critical for the prognosis of UCEC patients in a more precise way. This analytical method is also frequently used in other papers [35–37]. Patient risk scores were calculated and sorted from low to high. Then, they were divided into the high-risk group and low-risk group (Fig. 3A) based on the median. PCA demonstrated that the patients with different risks were already effectively dividing into two groups (Fig. 3C). In addition, we performed *t*-distributed Stochastic random neighbor embedding (TSNE) to validate the PCA results (Fig. 3D). The higher the risk score is, the higher the mortality in the high-risk group will be as compared to the low-risk group (Fig. 3B). The K-M survival analysis showed that the survival was statistically significant in both high-risk and low-risk groups. The survival rate was decreasing year by year over time. The survival rate of the high-risk group was significantly lower than that of the low-risk group (Fig. 3E). Single-gene survival analysis (Fig. 4A–G) was performed based on the expression profiles of the seven prognostic genes. The sensitivity and specificity of the prognostic model were evaluated using ROC analysis. It was found that the ROC area under curve of 1-, 2-, and 3-year survival rates was 0.701, 0.702, and 0.689 (Fig. 3F), respectively. It indicates that the risk model can better predict the patient survival condition. The heatmap showed the expression of prognosis-related NRLncRNAs between high-risk and low-risk groups (Fig. 4H).

### Independent Prognostic Value of Prognostic Risk Score Model

**Fig. 5:**
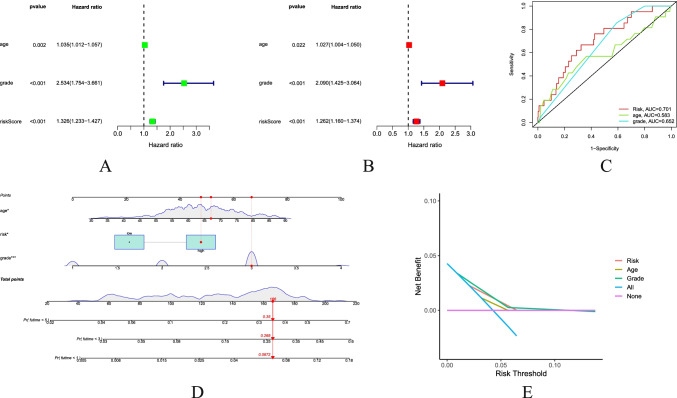
Combination of NRLncRNAs and clinical characteristics in predicting UCEC prognosis. **A** Univariate independent prognostic analysis. **B** Multivariate independent prognostic analysis. **C** The AUC of the ROC curve validated the prognostic accuracy of the prognosis-related clinical characteristics. **D** The associated nomogram for predicting the OS of patients with UCEC at 1, 3, and 5 years. **E** Decision curve analysis of associated clinicopathological features and risk signature

In order to evaluate whether the prognostic risk score model could be used as an independent prognostic predictor, the univariate Cox regression analysis and multivariate Cox regression analysis were performed. Both univariate Cox regression analysis and multivariate Cox regression analysis showed that the age, grades, and risk scores were significantly correlated with the OS in UCEC patients (Fig. 5A, B). Therefore, the age, grades, and risk scores can be used as independent prognostic factors for evaluating patients with UCEC. The ROC curve illustrated the accuracy of these indicators as the independent prognostic factors (Fig. 5C) in patients, where the age had a low accuracy as an independent prognostic factor. The nomograph was used to predict the results for each patient (Fig. 5D). Decision curves showed a greater sensitivity to risk scores and disease grades (Fig. 5E).

### Research Progress of NRG-Related Signaling Pathways

**Fig. 6:**
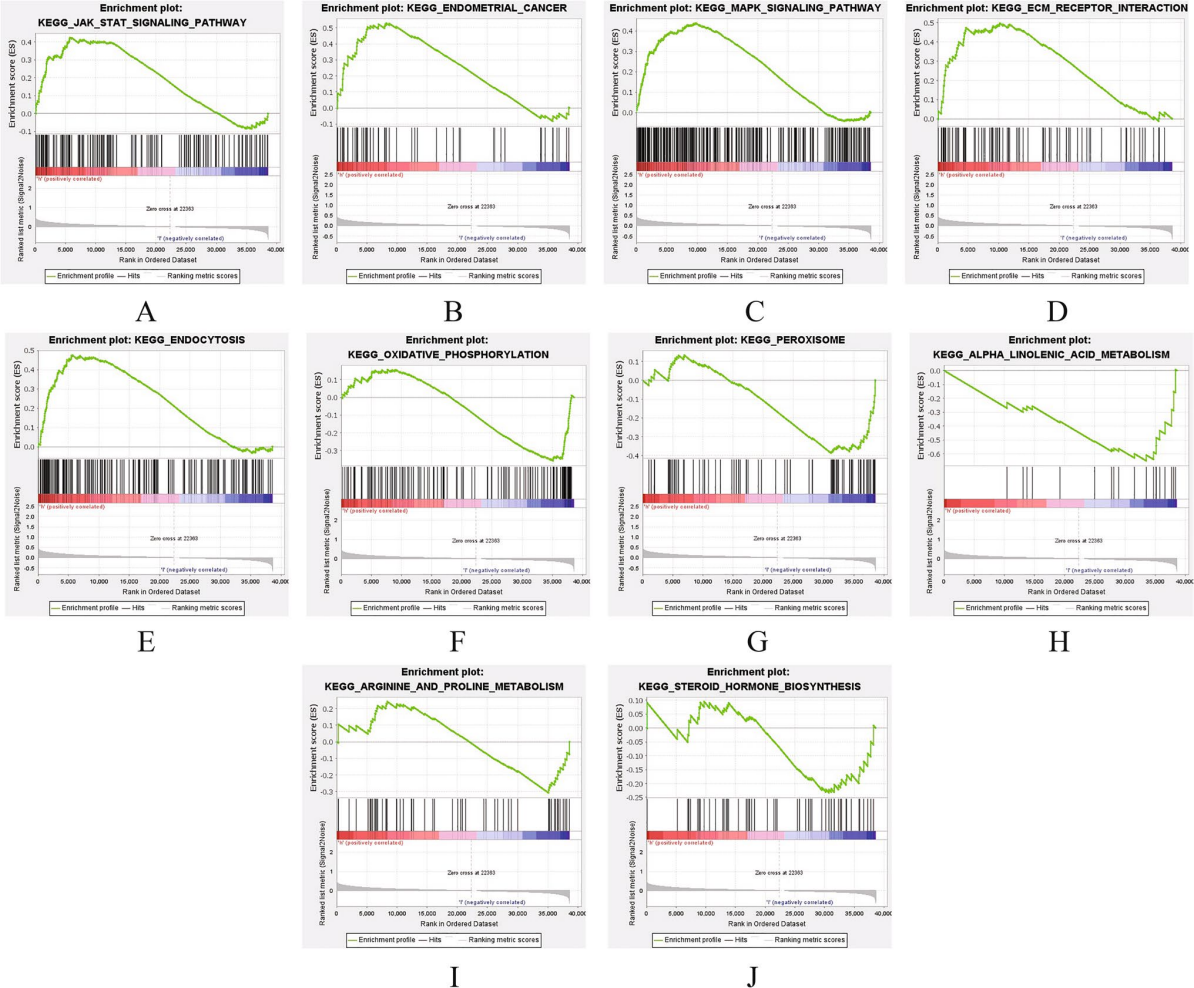
Gene set enrichment analysis (GSEA) based on the prognostic NRLncRNAs signature for high-risk group and low-risk group. **A**–**E** Five remarkably enriched active pathways in the high-risk group. **F**–**J** Five remarkably enriched active pathways in the low-risk group

In order to explore the biological function and signaling transduction pathways of LncRNAs associated with necroptosis, gene set enrichment analysis (GSEA) was performed using differentially expressed genes between the high-risk and low-risk groups. The results are shown in Fig. 6A–J. In the UCEC high-risk group, the endometrial cancer, jak stat signaling, MAPK signaling, ECM receptor, and endocytosis and so on related pathways were more active, while in the UCEC low-risk group, the peroxisome, steroid hormone biosynthesis, oxidative phosphorylation, arginine and proline metabolism, alpha linolenic acid metabolism, and so on related pathways were more active.

### Functional Analysis of Differential Genes in High-Risk and Low-Risk Groups

**Fig. 7:**
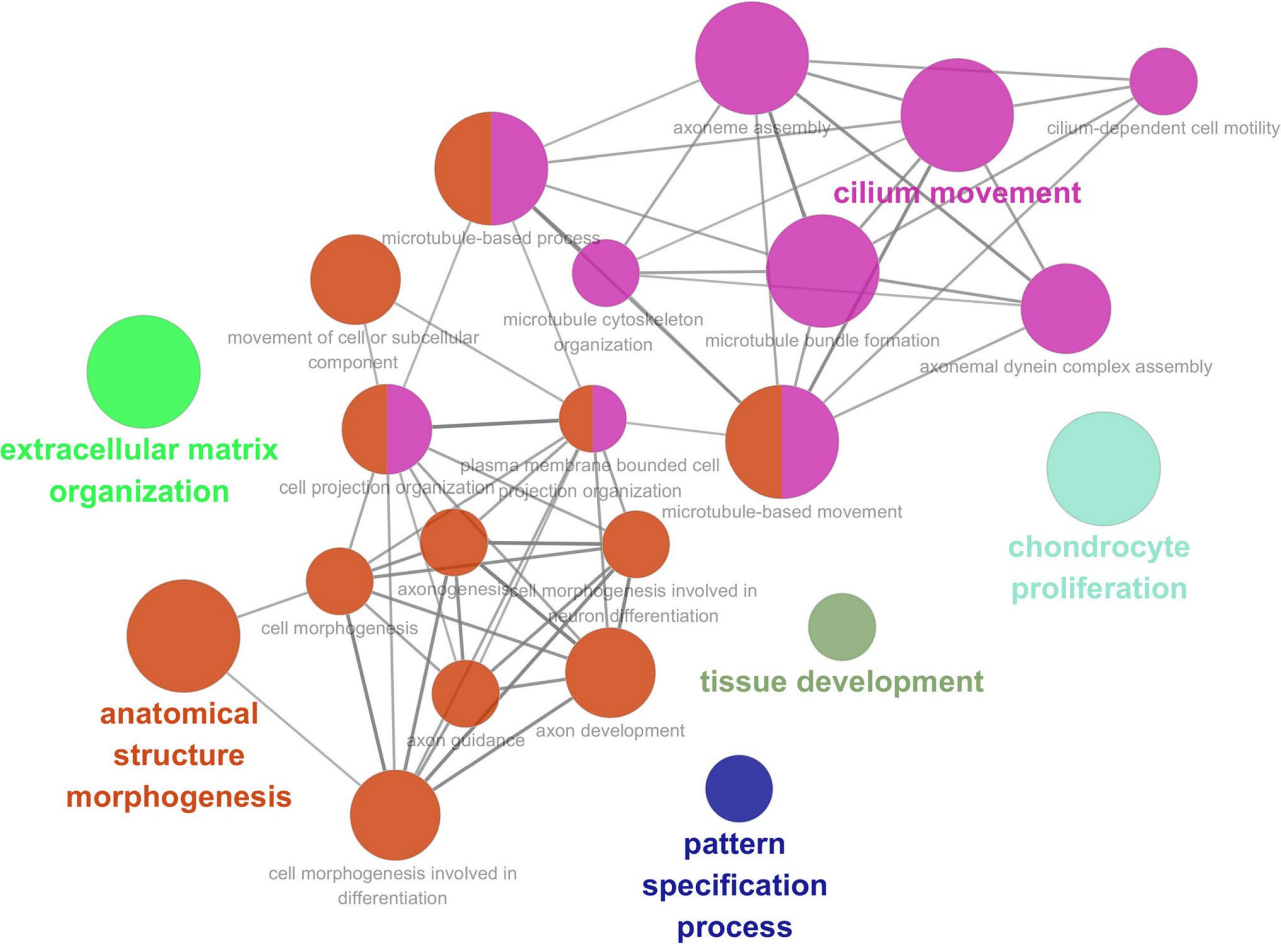
Functional analysis of differentially expressed genes between high-risk group and low-risk group

Functional analysis was carried out for those differentially expressed genes between the high-risk and low-risk groups using the ClueGO plugin in Cytoscape software (3.7.2), and the results of functional analysis are shown in Fig. 7. From the results, we found the “extracellular matrix organization,” “cilium movement,” “chondrocyte,” “tissue development,” “pattern specification process,” and “anatomical structure morphogenesis.” In addition, the complex network formed between the anatomical structure morphogenesis and cilium movement had closer interactions.

### Immune Infiltration Level and m6a Correlation Analysis

**Fig. 8:**
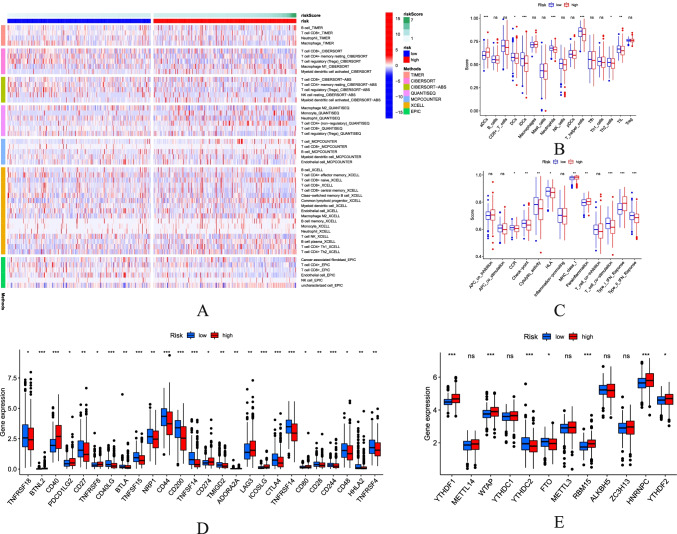
Potential role of risk signature in UCEC immune status and m6A-related genes. **A** The heatmap for immune responses based on TIMER, CIBERSORT, CIBERSORT-ABS, QUANTISEQ, MCPCOUNTER, XCELL, and EPIC between low-risk and high-risk group. **B** Comparison of ssGSEA scores of 16 types of immune cells between low-risk and high-risk group. **C** Comparison of the enrichment scores of 13 immune-related pathways between low-risk and high-risk group. **D** Expression of immune checkpoints between high-risk groups and low-risk groups. **E** Associations between the risk signature and m6A-related genes. *P* values mean: *P < 0.05; **P < 0.01; ***P < 0.001; ns not significant

Immuno-related heatmaps based on the TIMER, CiberSort, QUANTISEQ, MCP-Counter, XCell, and EPIC algorithms are shown in Fig. 8A. Given the key role of necroptosis in the immune process in human cells, especially in the tumor microenvironment, we compared the enrichment scores of 16 immune cells and the activity of 13 immune-related pathways between the low and high populations using a single-sample gene set enrichment analysis (SsGSEA) (Fig. 8B, C). The results showed that the difference in aDCs, DCs, iDCs, neutrophils, T helper cells, Th2 cells, TIL, CCR, check-points, cytolytic activity, HLA, MHC class I, parainflammation and T cell co-stimulation, type I IFN response, and type II IFN response was statistically significant between the two groups. In consideration of the importance of checkpoint-based immunotherapy, further differences (Fig. 8D) in the expression of immune checkpoints were discovered between the two groups (Fig. 8D). Due to the key role of necroptosis in the m6a process in human cells, the differences in the expression of m6a-related genes were found between the two groups (Fig. 8E).

## Discussion

In recent years, sequencing technology has gradually developed and progressed. LncRNA, CircRNA, and MirRNA have been detected [38–40]. Prognostic prediction of cancer by detecting the products of the sequencing technology contributes to the clinical treatment of patients [41, 42]. LncRNA, as an RNA with more than 200 nucleotides in length, the LncRNA may regulate the gene transfer rate and translational and post-translational modification [43]. The LncRNA is involved in processes such as proliferation, differentiation, migration, invasion, and apoptosis in cells [44]. A large number of studies have shown the important value of LncRNAs for the diagnosis and treatment of cancer [16, 45]. Therefore, this paper has investigated necroptosis-related LncRNAs to predict the survival of the patients with UCEC.

In the present study, we have screened the LncRNAs associated with both necroptosis and UCEC and constructed a prognostic model for the UCEC containing seven necroptosis-related LncRNAs. Moreover, this model has a good prediction for the patient survival after being validated by ROC curve [46]. The seven prognostic NRLncRNAs are PCAT19, CDKN2B-AS1, LINC01936, LINC02178, BMPR1B-DT, LINC00237, and TRPM2-AS, respectively. Among them, the high expression of TRPM2-AS, LINC01936, and LINC02178 represents a shorter survival time in patients with UCEC. While the high expression of PCAT19, CDKN2B-AS1, BMPR1B-DT, and LINC00237 represents a longer survival time in patients with UCEC.

Currently, many studies have confirmed that the above LncRNAs are important for the prognosis and progression of certain cancers. For example, LncRNA PCAT19 plays an important role [47] in the snp-mediated promoter-enhancer conversion mechanism in the process of regulating prostate cancer and adjusts the proliferation [48] of laryngeal cancer cells by regulating the miR-182/PDK4 axis. It has been shown that LncRNA CDKN2B-AS1 may act as a novel prognostic factor [49] in the immune microenvironment of UCEC. LncRNA LINC01936 can be an important factor [50] in the construction of a competitive endogenous RNA network related to the survival of patients with lung adenocarcinoma. LncRNA LINC02178 could also be used as a prognostic signature [51] in the prediction of the patients with bladder urothelial cancer. LncRNA BMPR1B-DT can also be used as one of the LncRNAs for predicting survival in patients with ovarian cancer to guide targeted medications [52]. LncRNA TRPM2-AS inhibits the cellular processes [53] such as glioma growth and invasion by JNK, c-Jun, and RGS4. Furthermore, according to Fig. 2F, we have found that LncRNA BMPR1B-DT and TRPM2-AS are co-expressed with the necroptosis-related gene TRAF5, as well as LncRNA PCAT19 with the necroptosis-related gene MOK, LncRNA LINC01936 with the necroptosis-related gene PKN1, LncRNA LINC02178 with the necroptosis-related gene CCL2, LncRNA LINC00237 with the necroptosis-related gene PCSK1, and LncRNA TRPM2-AS with the necroptosis-related genes MOK and REL. Genes associated with prognostic LncRNA have a great impact on cancer. For example, necroptosis-induced CCL2 release depends on the activation of the receptor interaction protein 1 (RIP1)/RIP3/mixed lineage kinase-like (MLKL) pseudokinase, which may promote the oncogenic phenotype [54] of tumor-associated astrocyte TAA.LncRNA. The identified hits need further experimental validation and methods for validation.

In addition, the expression of p53 overexpression has a significant correlation with prognostic markers. The study demonstrated that PCAT19 negatively regulates the p53 tumor-suppression pathway, promoting cancer cell proliferation in patients with NSCLC [55]. Downregulated LncRNA TRPM2-AS induced cell apoptosis and altered cell cycle distribution through activating the p53-p66shc pathway [56]. The expression of the CDKN2B-AS1 and adjacent gene, CDKN2A, are downregulated in the peripheral blood of patients with IPF, which activates the p53-signaling pathway to promote lung cancer formation [57]. The above arguments suggest that these prognostic markers may also play an important role in regulating the prognosis of endometrial cancer.

Furthermore, we have detected that the relevant pathways are more active in the high-risk group via GSEA analysis, including MAPK signaling pathway, Jak-STAT signaling pathway, and ECM-receptor interaction. MAPK signaling pathway is closely related to numerous tumors [58–60]. Besides, this pathway is involved in the regulation of necroptosis [61, 62] to some extent. However, the Jak-STAT signaling pathway partly suppresses the colorectal cancer cell proliferation and stimulated apoptosis [63]. ECM-receptor interaction may promote [64] proliferation and invasion in some cancer cells. However, we have discovered that peroxisome, steroid hormone biosynthesis, and oxidative phosphorylation pathways are more active in the low-risk group. The peroxisome pathway is involved in most oncogenic processes [65, 66]. Oxidative phosphorylation is involved in inducing the necrosis [67, 68] of various cells. The steroid hormone biosynthesis pathway then affects the progression [69] of UCEC. This may be associated with the function of cellular pattern specification process, anatomical structure morphogenesis, and tissue development (Fig. 7).

Through the study, we have also found out a different expression of immune-related factors in the high-risk and low-risk groups. Antibody drug conjugates (ADCs) are exciting types of tumor therapy [70]. In the present study, ADCs are active in the high-risk group. Perhaps the inhibition of ADCs is a new direction for tumor treatment in the future. In recent years, blocking T helper cells by targeted regulation to treat cancer has become a trend [71]. These hosts with strong immunocompetence will select cancer cells with less immunogenicity during tumor development to escape the antitumor immune response [72]. Furthermore, immune checkpoint-related genes are clearly differentially expressed in both high-risk and low-risk groups, generally highly expressed in the high-risk group. Moreover, although the detailed mechanisms behind these relationships require further investigation and validation (Fig. 8E), our necroptosis-associated LncRNA signatures could effectively predict the expression levels of m6A-related genes.

### Limitations

Despite the strong predictive value in the established risk model, there are some shortcomings in our current study. Firstly, the original datasets for LncRNA-related models are only retrieved from the TCGA database. The number of control patients used in the study is small compared to the tumor sample group (23 vs 552). The reliability and accuracy of our risk model require to be validated in other external datasets and large-scale clinical cohorts. Secondly, the significant shortcomings in this study are the lack of experimental validation of the identified targets. The mechanism by which necroptosis regulates the exact process of UCEC is unclear. It needs to be elucidated by further experimental studies.

## Conclusion

This study has identified seven necroptosis-related LncRNA signatures for the first time. It provides a valuable basis for a more accurate prognostic prediction.

## Supplementary Information

ESM 1 (ZIP 4.0 KB)

ESM 2 (ZIP 47.6 MB)

## Data Availability

All the data in this paper support the results of this study.
